# Comodulation of NO-Dependent Vasodilation by Erythroid Band 3 and Hemoglobin: A GP.Mur Athlete Study

**DOI:** 10.3389/fcvm.2021.740100

**Published:** 2021-11-29

**Authors:** Kate Hsu, Yen-Yu Liu, Wei-Chin Tseng, Kuang-Tse Huang, Chia-Yuan Liu, Li-Yang Chen, Hui-Lin Lee, Hui-Ju Lin, Kuo-Wei Tseng, Hung-I Yeh

**Affiliations:** ^1^Department of Medical Research, MacKay Memorial Hospital, New Taipei City, Taiwan; ^2^MacKay Junior College of Medicine, Nursing, and Management, New Taipei City, Taiwan; ^3^Institute of Biomedical Sciences, Mackay Medical College, New Taipei City, Taiwan; ^4^Department of Critical Care Medicine, MacKay Memorial Hospital, New Taipei City, Taiwan; ^5^Department of Physical Education, University of Taipei, Taipei, Taiwan; ^6^Department of Chemical Engineering, National Chung-Cheng University, Chia-Yi, Taiwan; ^7^Division of Gastroenterology, Department of Internal Medicine, MacKay Memorial Hospital, Taipei, Taiwan; ^8^Department of Medicine, MacKay Medical College, New Taipei City, Taiwan; ^9^Department of Exercise and Health Sciences, University of Taipei, Taipei, Taiwan; ^10^Division of Cardiology, Departments of Internal Medicine, MacKay Memorial Hospital, Taipei, Taiwan

**Keywords:** flow-mediated vasodilation (FMD), nitric oxide (NO), Band 3, GP.Mur, red blood cell (RBC), hemoglobin (Hb), nitrite, athletes

## Abstract

GP.Mur, a red blood cell (RBC) hybrid protein encoded by *glycophorin B-A-B*, increases expression of erythroid band 3 (Anion Exchanger-1, SLC4A1). GP.Mur is extremely rare but has a prevalence of 1–10% in regions of Southeast Asia. We unexpectedly found slightly higher blood pressure (BP) among healthy Taiwanese adults with GP.Mur. Since band 3 has been suggested to interact with hemoglobin (Hb) to modulate nitric oxide (NO)-dependent hypoxic vasodilation during the respiratory cycle, we hypothesized that GP.Mur red cells could exert differentiable effects on vascular tone. Here we recruited GP.Mur-positive and GP.Mur-negative elite male college athletes, as well as age-matched, GP.Mur-negative non-athletes, for NO-dependent flow-mediated dilation (FMD) and NO-independent dilation (NID). The subjects were also tested for plasma nitrite and nitrate before and after arterial occlusion in FMD. GP.Mur+ and non-GP.Mur athletes exhibited similar heart rates and blood pressure, but GP.Mur+ athletes showed significantly lower FMD (4.8 ± 2.4%) than non-GP.Mur athletes (6.5 ± 2.1%). NO-independent vasodilation was not affected by GP.Mur. As Hb controls intravascular NO bioavailability, we examined the effect of Hb on limiting FMD and found it to be significantly stronger in GP.Mur+ subjects. Biochemically, plasma nitrite levels were directly proportional to individual band 3 expression on the red cell membrane. The increase of plasma nitrite triggered by arterial occlusion also showed small dependency on band 3 levels in non-GP.Mur subjects. By the GP.Mur comparative study, we unveiled comodulation of NO-dependent vasodilation by band 3 and Hb, and verified the long-pending role of erythroid band 3 in this process.

## Introduction

Hemoglobin (Hb) modulates vascular tone by controlling nitric oxide bioavailability in the blood stream. The chemical reaction between Hb and NO metabolites is coupled with the dynamics of O_2_ saturation and desaturation of Hb (1–5). While intraerythrocytic Hb is considered the most powerful NO scavenger in human body, its effect on NO-associated vasodilation is limited by the RBC membrane (6). Hb-mediated NO bioavailability has been proposed to require band 3, the main anion transporter on RBCs. The interaction between band 3 and deoxygenated Hb in the submembranous domain has been suggested to assist the transport of NO metabolites (e.g., nitrite and SNO) across the red cell membrane by unclear means (7–10). Currently, the role of band 3 in RBC-dependent NOx processing and hypoxic vasodilation is enigmatic.

GP.Mur (also known as Miltenberger subtype III or Mi.III), is a Southeast Asian special blood type with 1–10% prevalence among Filipinos, Vietnamese, Thai, and Taiwanese, but is very rare among Caucasian (0.0098%), northern Asian like Han Chinese and Japanese (0.006%) (11–14). Biochemically, GP.Mur is a hybrid protein originated from homologous gene recombination of the highly homologous *GPYB* and *GYPA* genes (15). As GP.Mur contains a segment of glycophorin A (GPA) and GPA acts as a chaperone to band 3 (16, 17), the consequence of the expression of GP.Mur is to support increased expression of band 3 on the erythrocyte surface (18). Band 3, a Cl^−^/${\text{HCO}}_{3}^{-}$ antiporter, allows ${\text{HCO}}_{3}^{-}$ permeation across the erythrocyte membrane, which is physiologically essential for intraerythrocytic CO_2_/${\text{HCO}}_{3}^{-}$ conversion and postnatal CO_2_ respiration (19–21). People with GP.Mur blood type express more band 3 on RBCs and show faster CO_2_ respiration after a mild physical challenge (18, 22). Unexpectedly, from our previous human exercise study on Taiwanese adults aged up to 50 years old, GP.Mur+ subjects exhibit slightly higher blood pressure than the non-GP.Mur counterparts (22, 23).

The structural-functional hallmarks of GP.Mur+ red cells are enhanced band 3 expression (18) and altered band 3 complex structure with other membrane proteins on the red cell surface (21, 24–26). GP.Mur+ red cells present stronger band 3-AQP1 interaction (18, 26), contain reduced amount of RhAG and consequently altered band 3-Rh/RhAG macrocomplex organization (25, 27), and are more resilient physically in the osmolarity-fragility test (18). These presumably could affect band 3 interaction with submembranous deoxy Hb and metHb (28) and/or result in differential NOx processing, vasodilation, and blood pressure setting. To test this hypothesis, we examined NO-dependent FMD and NO-independent vasodilation primarily in a uniform cohort—Taiwanese elite college athletes who have been trained professionally for national and/or international sports competitions for approximately half of their lifetime. Together with blood measurements for band 3 and NOx changes during FMD, we identified co-modulation of FMD by erythroid band 3 and Hb.

## Materials and Methods

### Subject Recruitment, GP.Mur Phenotyping, and Study Design

This human study protocol had been approved by MMH-Institutional Review Board (IRB registration: 19MMHIS081e). For uniform cohorts, we primarily recruited male elite athletes from top sports universities in Taiwan, where they enrolled with their competition scores from national/international games. Before college, all of them had professional sports training and competition experiences for at least 6 years. All subjects were free from major illness. To maintain eligibility for sports competitions, they were prohibited from taking most medication. We also recruited age- and BMI-matched, healthy male non-athletes as controls. All subjects had given written informed consents before participating in this study.

GP.Mur blood type was first determined by red cell serology using anti-Mur and anti-Mi^a^ antisera, and then confirmed by PCR-sequencing, as described (29). It has long been observed in Taiwan that the prevalence of GP.Mur among elite Taiwanese athletes is much higher. We pre-screened 129 elite male college athletes and found 32 of them with GP.Mur blood type (24.8%). Among the elite athletes, 51 of them agreed to participate in this study, and 19 of them bear GP.Mur phenotype (37.3%). As for the non-athlete subjects, only one out of 20 bear GP.Mur (5%), which is similar to the occurrence frequency of GP.Mur in general Taiwanese population.

**Figure 1:**
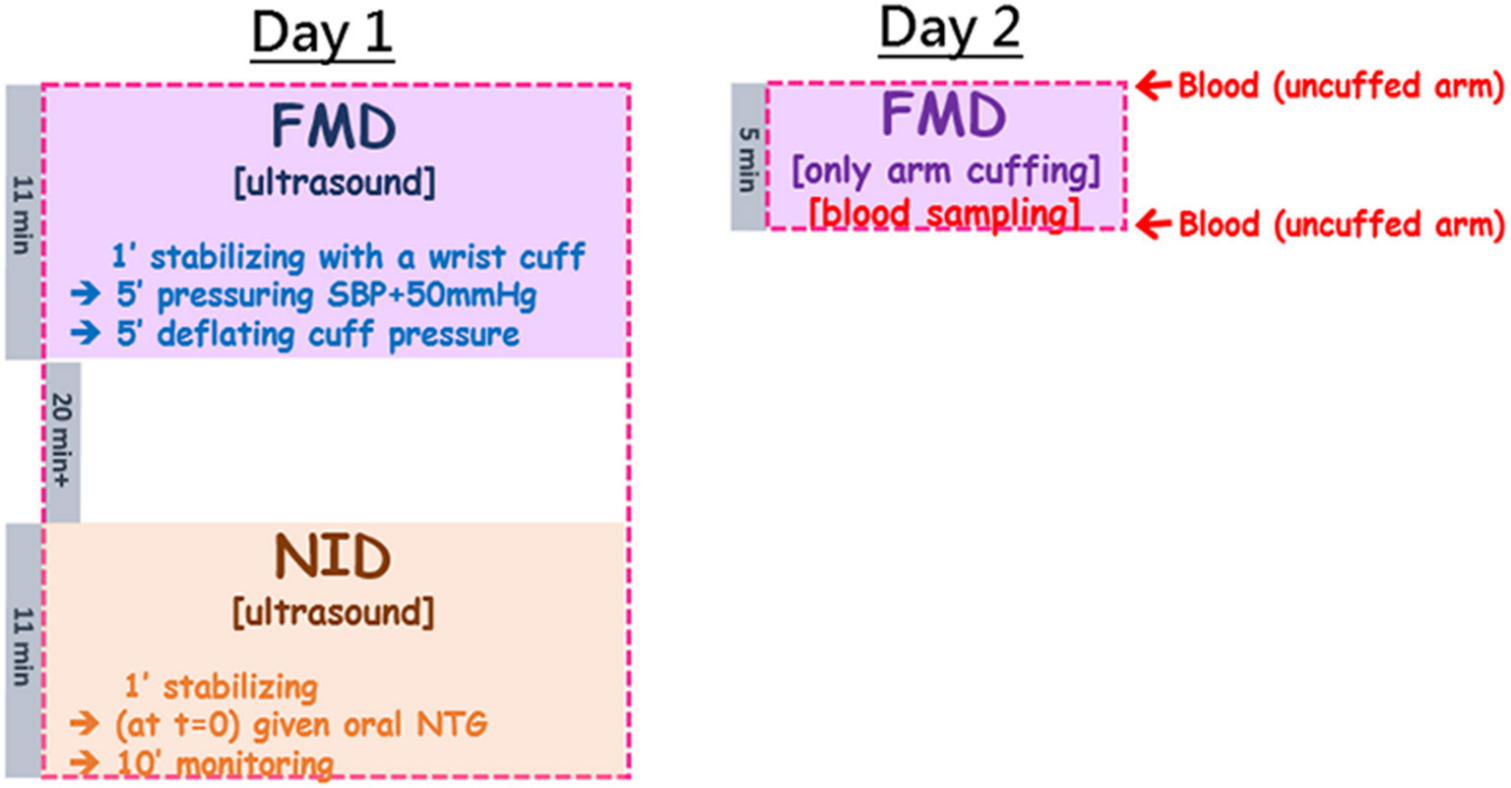
The study flow chart combined ultrasound measurements of FMD and NID, and blood sampling before cuffing and right upon pressure deflation which allowed assessment of plasma NOx changes by arterial occlusion during FMD. The study was arranged in two separate days: Day 1 for ultrasound-based tests; Day 2 for blood sampling before and after 5-min brachial arterial constriction. In Day 1, the subject was started with the ultrasound measurement for FMD, followed by a minimal 20-min break to allow recovery of the tested brachial artery, and then the second ultrasound measurement for NID. FMD took ~11 min (including 1 minute of stabilization, 5 min of cuff pressuring to 50 mmHg above the subject's SBP, and another 5 min of observation after wrist cuff deflation). The NID measurement also began with 1-min stabilization, followed by oral administration of NTG and then 10-min monitoring of the changes of the brachial arterial diameter. In Day 2, the setting was very similar to FMD in Day 1 (5 min of pressuring to 50 mmHg above the subject's SBP), but ultrasound was replaced by blood sampling before and after cuffing.

The study design was illustrated in the flow chart in Figure 1. Briefly, each subject was tested on two separate days: 1 day for ultrasound measurements of FMD and then NID; the other day for assessments of the changes of blood plasma NO metabolites during FMD (blood withdrawal mostly completed within 1 min after cuff deflation). The subjects were not allowed intake of food or caffeinated drinks at least 2 h prior to FMD tests on both days. They were asked not to practice sports at least 6 h before testing.

### Ultrasound Measurements for FMD and NID

We followed the FMD and NID protocols provided by the Hitachi-Aloka eTRACKING (echo-tracking) system (30). The Aloka eTRACKING program was installed in the Hitachi Arietta 60 (Ar60) ultrasound system equipped with a 12–2 MHz transducer (L441).

**Figure 2:**
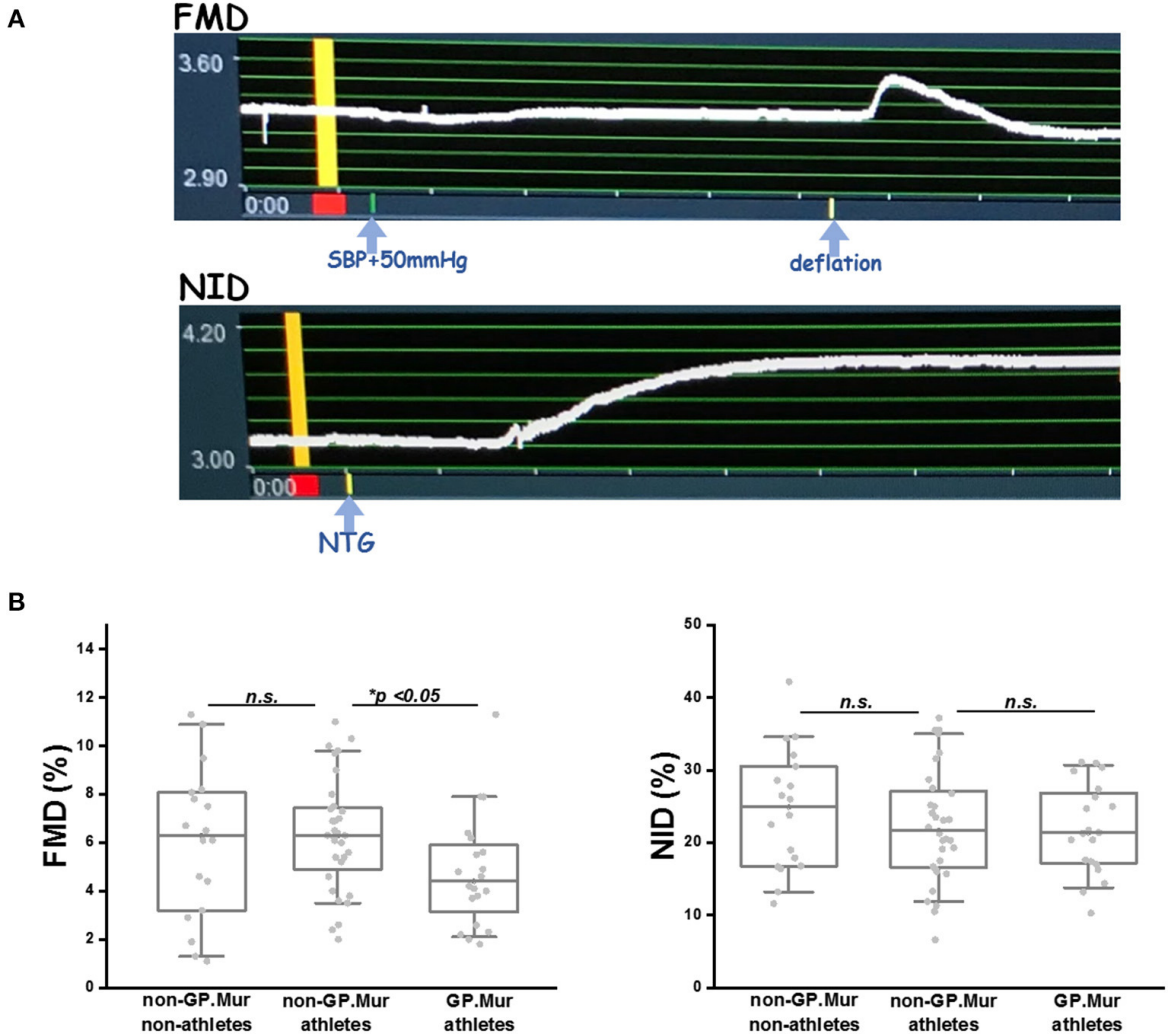
FMD was significantly lowered in GP.Mur+ male elite athletes than GP.Mur-negative athletes or nonathletes, whereas NID was not affected by GP.Mur expression. **(A)** TOP: An example of FMD captured by the eTRACKING program of Hitachi-Aloka ARIETTA 60. The diameter of the region tracked is shown at the y-axis (unit: mm). The recorded time is shown at the x-axis (unit: minute). The yellow bar indicates the baseline of the vessel diameter that was to be compared with the maximally dilated diameter of the same region for %FMD or %NID. BOTTOM: An example of NO-independent vasodilation. **(B)** FMD was compared among the three groups of subjects—GP.Mur athletes, non-GP.Mur athletes, and non-athletes. Each dot represented the datum of a subject. The box covered data ranging 25–75%; the line inside a box indicated the median value. Data between the paired groups were compared by two-sample *t*-test (*p < 0.05). RIGHT: Comparison of NID (*n. s*., not significant).

Before ultrasound examination, each subject was allowed to calm down in bed in a dark, quiet examination room, and his left wrist was wrapped with a pneumatic cuff. The L441 transducer locked in a probe holder was placed about 5–10 cm above his left elbow to image his left brachial artery. Under the FMD mode of eTRACKING, a selected region of his brachial artery was first focused and diameter changes in the focused brachial artery were recorded over time. The FMD recording began with 1-min baseline (stabilization), 5-min inflation of the wrist pressure to 50 mmHg above his systolic blood pressure (SBP), followed by deflation and 5 more minutes of monitoring the changes of the brachial arterial diameter. The peak of reactive hyperemia generally appeared within 30–90 s post-arterial occlusion, and the brachial arterial diameter generally returned to its baseline level within 2 min (see Figure 2A).

After FMD, the subject was given a 30-min break and then stabilized again in bed for ultrasound examination of NID on a similar region of the brachial artery as for FMD. NO-independent dilation recording by eTRACKING also began with 1-min baseline. The subject was then given 0.6 mg sublingual nitroglycerin (Nitrostat®, *Pfizer*), and his changes of the brachial arterial diameter were recorded for 10 min. These subjects generally reached maximal vasodilation by the 6th minute post-nitroglycerin. Nitroglycerin-induced hyperemia generally persisted till the end of the total 11-min recording (see Figure 2A).

### Determination of Blood Plasma Nitrate and Nitrite Concentrations

To find out blood plasma NOx changes during FMD, on a separate visit the subject underwent a similar mechanical stimulus–5-min cuff inflation to 50 mmHg above his SBP on his right upper arm. Venous blood was sampled from his left median cubital vein at two timepoints: (1) before cuffing; (2) after deflation of the arm cuff. This allowed us to evaluate the consequence of regional ischemia and onset of NO-dependent vasodilation reflected by plasma NOx. Our blood sampling protocol for NOx during reactive hyperemia followed a report by Allen et al. (31), with slight modification.

Right after blood withdrawal into an ACD tube, the sample was immediately centrifuged to separate plasma from RBCs. Blood plasma was further filtered using an Amicon Ultra-4 Centrifugal unit with 10-kDa cutoff at 3,220 × *g* for 30 min at 4°C, and then aliquoted and stored at a −80°C freezer. Due to the short lifetime of nitrite, the handling process from blood withdrawal to sample storage in −80°C was limited to 45 min or less. Plasma NOx and ${\text{NO}}_{3}^{-}$ were quantitated using the Nitrate/Nitrite colorimetric assay kit (Cayman Chemical, MI, USA); plasma ${\text{NO}}_{2}^{-}$ was quantitated with the Nitrate/Nitrite fluorometric assay kit (Cayman).

### Band 3 Quantitation

RBCs were hypotonically ruptured to obtain the membrane fraction (ghost). Total proteins in freshly lysed ghosts were quantified by Lowry protein assay (Bio-RAD, CA, USA), and band 3 levels measured by band 3 sandwich ELISA. The band 3 sandwich ELISA was developed in house. Briefly, anti-band 3 rat monoclonal BRAC 18 (International Blood Group Reference Laboratory [IBGRL], NHS, UK) was applied as the coating antibody (1:500 dilution) onto an ELISA plate overnight at 4°C. RBC ghost lysates were then reacted with the coated BRAC 18 for 30 min at 37°C, followed by PBST (0.05% Tween-20-supplemented PBS) washes and incubation with another anti-band 3 mouse monoclonal BRIC 170 (IBGRL; 1:500 dilution) for 2 h at 37°C. The signal of BRIC 170-sample binding was amplified by incubation with 1:5,000 HRP-conjugated, anti-mouse-specific secondary IgG (Jackson ImmunoResearch Laboratories, PA, USA); this secondary anti-mouse antibody had been immunoaffinity-purified to yield minimal cross reactivity with rat. The standard curve for absolute quantitation of band 3 in the sandwich ELISA used a custom-made band 3 peptide that contains the known epitope sequence of BRIC 170 (AA 368-382); the custom-made peptide sequence is NGGPDDPLQQTGQLFGGLVR (AA 365-384 of human band 3 ([NP_000333.1]). One's band 3 level relative to all RBC membrane proteins in his sample (w/w) was obtained by dividing the band 3 concentration by the ghost protein concentration and expressed in percentile (%).

## Results

To probe into the potential impacts of GP.Mur/band 3 on NO-dependent reactive hyperemia in a uniform cohort, this study recruited primarily male elite college athletes from top-ranking Taiwan sports universities where the prevalence of GP.Mur is much higher than the general Taiwanese population. The athlete subjects all have similar age, lifestyle, and enrolled in rigorous sports training programs aiming for national/international competitions for approximately half of their lifetimes (Table 1). They were divided into GP.Mur-positive (19/70) and GP.Mur- negative athlete groups (32/70). For comparison, twenty age- and BMI-matched non-athlete subjects were also recruited (only 1/20 GP.Mur+). Compared to the non-GP.Mur non-athletes (19/70), both groups of athletes showed similarly lower heart rates and slightly higher systolic blood pressuer (Table 1).

**Table 1:** Subject characteristics.

**Group (*N*)**	**Non-GP.Mur non-athlete (19)**	**Non-GP.Mur athlete (32)**	**GP.Mur athlete (19)**	**Difference between athletes with GP.Mur and without**	**Difference between nonGP.Mur athletes and non-athletes**
Age (yrs)	23.6 ± 1.7	20.8 ± 3.0	22.2 ± 3.5	*n.s*.	*n.s*.
BMI	22.8 ± 2.6	24.7 ± 4.2	23.8 ± 2.8	*n.s*.	*n.s*.
% have smoked	5.3	31.3	26.3		
Athletic training yrs	0	10.4 ± 3.7	11.2 ± 3.6	*n.s*.	
Athletic training frequencies (hrs/wk)	0	15.0 ± 5.2	17.5 ± 9.9	*n.s*.	
*Distribution of sports specialties*		*71% mixed*	*67% mixed*		
		*23% sprint*	*22% sprint*		
		*6% endurance*	*11% endurance*		
Quiet heart rate (beats/min)	65.5 ± 10.8	59.5 ± 9.7	59.1 ± 6.8	*n.s*.	*p <0.05*
SBP (mmHg)	120.2 ± 8.8	127.9 ± 9.3	123.8 ± 13.4	*n.s*.	*p <0.05*
DBP (mmHg)	71.8 ± 8.1	66.7 ± 6.9	69.7 ± 8.3	*n.s*.	*n.s*.
Pulse pressure (mmHg)	48.4 ± 7.3	59.2 ± 8.4	54.1 ± 13.0	*n.s*.	*p <0.001*
RBC (10^∧^6/μL)	4.8 ± 0.7	5.0 ± 0.7	4.9 ± 0.4	*n.s*.	*n.s*.
Hb (g/dL)	15.1 ± 0.8	15.0 ± 1.0	14.9 ± 0.8	*n.s*.	*n.s*.
Hemotocrit (%)	45.1 ± 2.3	44.5 ± 2.6	44.2 ± 2.4	*n.s*.	*n.s*.
Platelets (10^∧^3/μL)	243.0 ± 43.7	229.5 ± 72.7	236.3 ± 68.0	*n.s*.	*n.s*.

*Data were shown in mean ± SD. BMI, body mass index. Mixed sports types here include judo, taekwondo, karate, soccer, basketball, wrestling, and soft tennis. The sprint athletes here are specialized in discus throw, shot put, hammer throw, javelin, high jump, weightlifting, 100/200 m run, and 50/100 m swim. The endurance athletes here are both mid-to-long distance runners. Data were shown in mean ± SD. Statistical differences were calculated by two-sample t-test; p <0.05 was deemed significant; n.s., not significant*.

### Lower FMD in GP.Mur Subjects

Compared to GP.Mur-negative athletes, FMD measured by ultrasound e-TRACKER was significantly lowered in GP.Mur+ athletes (4.6 ± 2.3% [GP.Mur+ athletes] vs. 6.3 ± 2.4% [non-GP.Mur athletes], ^*^p < 0.05), despite that their blood pressure profiles were not different (Figure 1, Tables 1, 2). Both GP.Mur+ and non-GP.Mur athlete groups exhibited similar degrees of NO-independent vasodilation triggered by nitroglycerin (Figure 2). The similar NID responses between GP.Mur+ and non-GP.Mur hint similar maximal functional capacities in their eNOS and smooth muscle cells (SMCs) (32). As for the GP.Mur-negative non-athletes, their FMD or NID were indistinguishable from either athlete groups (Figure 2, Table 2).

**Table 2:** FMD, NID, blood plasma NO metabolites, and band 3 levels on RBCs.

**Group (*N*)**	**Non-GP.Mur non-athlete (19)**	**Non-GP.Mur athlete (32)**	**GP.Mur athlete (19)**	***Difference between athletes with GP.Mur and without***	***Difference between nonGP.Mur athletes and non-athletes***
% FMD	6.0 ± 3.1	6.3 ± 2.4	4.6 ± 2.3	*p <0.05*	*n.s*.
% NID	24.5 ± 8.4	22.3 ± 7.9	21.4 ± 6.0	*n.s*.	*n.s*.
FMD baseline diameter (mm)	3.7 ± 0.5	3.9 ± 0.5	4.0 ± 0.4	*n.s*.	*n.s*.
NID baseline diameter (mm)	3.8 ± 0.6	3.8 ± 0.5	4.0 ± 0.4	*n.s*.	*n.s*.
Time to reach max diameter after deflation (s)	48.6 ± 10.6	57.0 ± 16.1	49.8 ± 15.7	*n.s*.	*p <0.05*
Plasma NOx before cuffing (μM)	9.63 ± 6.70	5.66 ± 3.52	6.02 ± 4.73	*n.s*.	*p <0.05*
Plasma NOx upon cuff deflation (μM)	9.13 ± 6.21	5.43 ± 3.64	5.87 ± 4.67	*n.s*.	*p <0.05*
Plasma ${\text{NO}}_{2}^{-}$ before cuffing (μM)	0.42 ± 0.15	0.47 ± 0.14	0.42 ± 0.16	*n.s*.	*n.s*.
Plasma ${\text{NO}}_{2}^{-}$ upon cuff delfation (μM)	0.45 ± 0.18	0.53 ± 0.20	0.46 ± 0.17	*n.s*.	*n.s*.
Plasma ${\text{NO}}_{3}^{-}$ before cuffing (μM)	9.21 ± 6.72	5.19 ± 3.55	5.60 ± 4.84	*n.s*.	*p <0.05*
Plasma ${\text{NO}}_{3}^{-}$ upon cuff delfation (μM)	8.68 ± 6.22	4.91 ± 3.70	5.41 ± 4.78	*n.s*.	*p <0.05*
Δ[${\text{NO}}_{2}^{-}$]_cuffing_ (μM)	0.03 ± 0.09	0.07 ± 0.08	0.05 ± 0.09	*n.s*.	*n.s*.
Δ[${\text{NO}}_{3}^{-}$]_cuffing_ (μM)	−0.24 ± 0.58	−0.19 ± 0.40	−0.23 ± 0.33	*n.s*.	*n.s*.
% band 3 in RBC membrane proteins (w/w)	4.15 ± 1.89	4.30 ± 2.41	5.22 ± 1.69	*p <0.05*	*n.s*.

### FMD Limited More by Hb in GP.Mur+ Than in Non-GP.Mur Subjects

**Figure 3:**
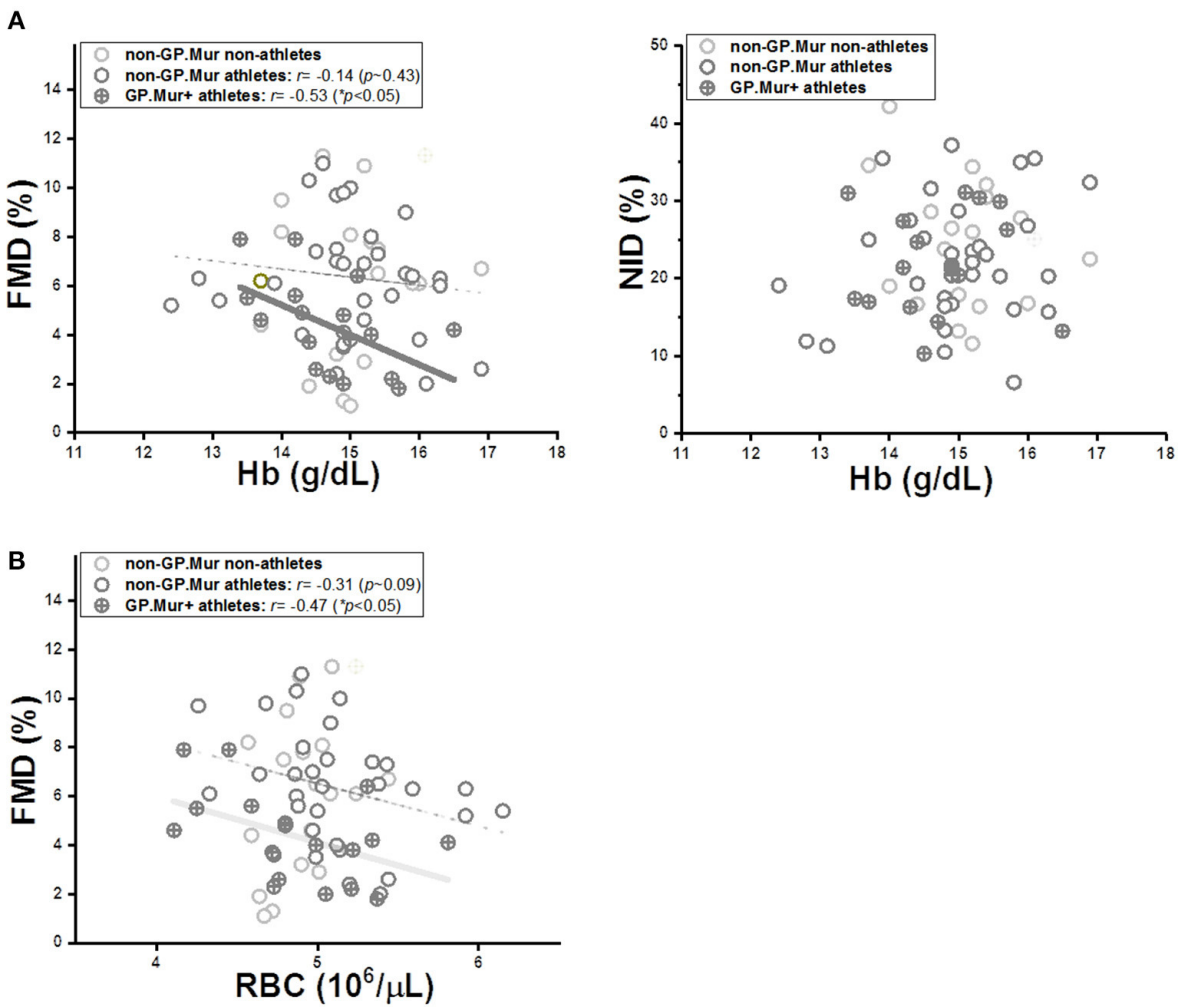
The limiting effect of intraerythrocytic Hb on FMD was more significant in GP.Mur+ athletes (cross circular symbols) than in the non-GP.Mur control subjects (empty circular symbols). **(A)** LEFT: Linear regression analyses revealed a significant inverse correlation between Hb and FMD in GP.Mur+ athletes, which was less obvious in non-GP.Mur athlete and non-athlete subjects (GP.Mur: Pearson's *r* = −0.53 (*p < 0.05); non-GP.Mur: weak correlation). RIGHT: Intraerythrocytic Hb exerted no specific effects on NID for GP.Mur+ or non-GP.Mur subjects. **(B)** The dependency of FMD on RBC count was similar for both GP.Mur+ and non-GP.Mur groups. Solid thick lines represent significant linear fitting; thin, dotted lines represent poor fitting (*p* > 0.05).

In a study on hypertensive patients with high-altitude erythropoiesis, FMD in these patients was inversely correlated with their abnormally high Hb (>21 g/dL) and excessive number of circulating RBC cells (33). Here, our subjects were healthy young students with Hb and RBC count in the normal ranges. Their Hb and RBC count were not different because of GP.Mur (Table 1). But only in GP.Mur+ subjects was FMD significantly inversely correlated with Hb (Figure 3A: thick solid line). This trend would not be obvious in most non-GP.Mur people with normal Hb, as shown with the poor fitting in Figure 3A (33). If comparing the Pearson's correlation *r* between Hb and FMD, the correlation in GP.Mur was 4-fold that in non-GP.Mur (−0.53 [GP.Mur+] vs. −0.14 [non-GP.Mur]) (Figure 3A). From the data, Hb in GP.Mur+ RBCs was more effective in scavenging NO, and this likely involved distinct components in the GP.Mur red cell membrane. In contrast, the correlation between Hb and NID was poor for all three groups (*p* > 0.05), indicating that NID was not associated with Hb (Figure 3A, right). These results confirm the central role of Hb in NO-dependent vasodilation.

On the other hand, FMD correlation with RBC count was not much different between GP.Mur and non-GP.Mur (Figure 3B: *r* or the slopes for GP.Mur+ athletes, −0.47; for non-GP.Mur athletes, −0.31). From their similar dependency of FMD on RBC count, their drastically different FMD sensitivity to Hb had to be primarily triggered by their differences in the RBC membrane composition (i.e., GP.Mur/band 3 protein complexes).

### Blood Plasma Nitrite Levels Dynamically Correlated With Band 3 Expression

**Figure 4:**
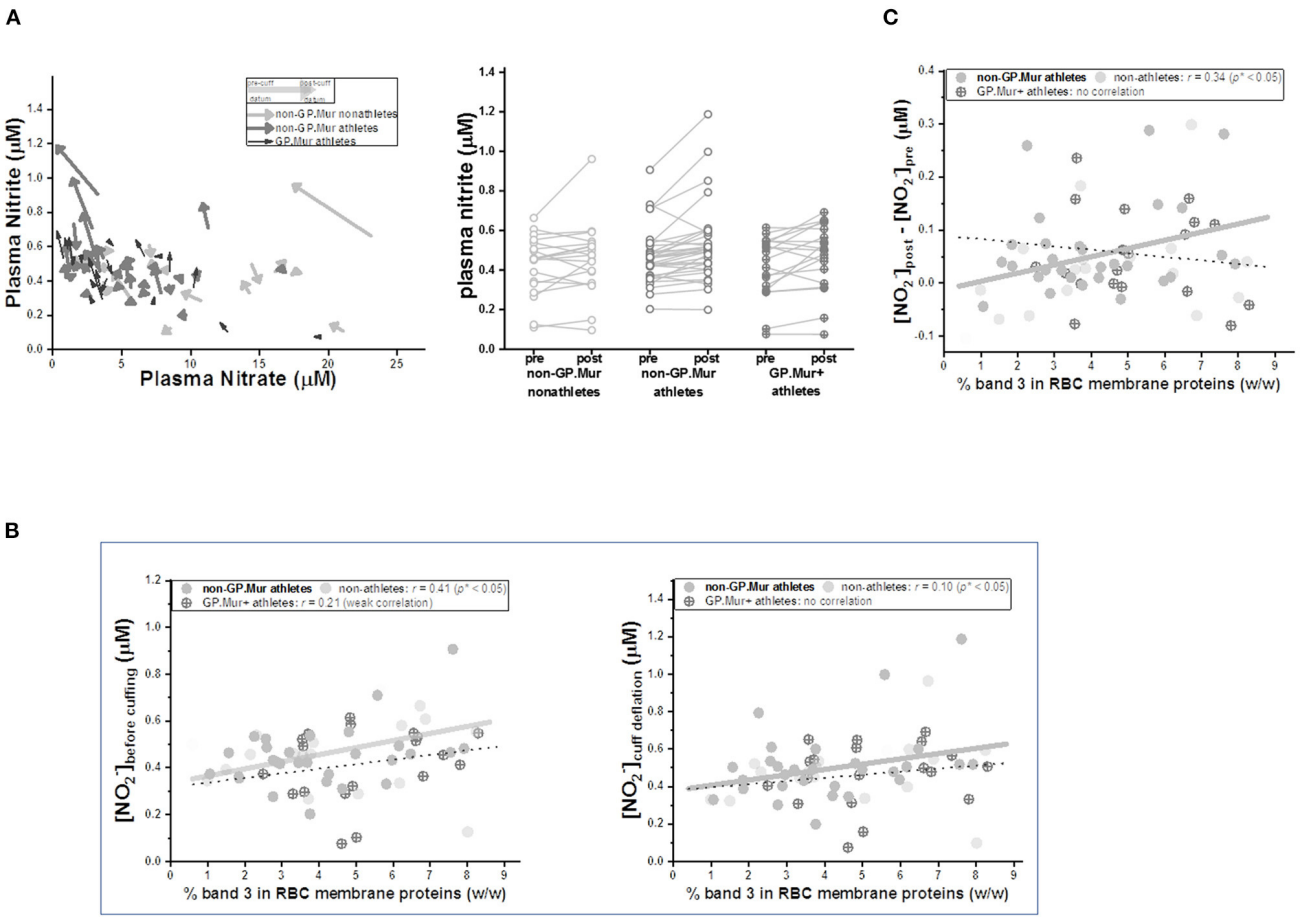
Arterial occlusion following cuff deflation during FMD decreased plasma nitrate and increased nitrite, the latter of which was quantitatively directly correlated with individual erythroid band 3 levels. **(A)** LEFT: Plasma nitrite and nitrate levels before cuffing and upon deflation were compared. Their changes (Δ[${\text{NO}}_{2}^{-}$] and Δ[${\text{NO}}_{3}^{-}$]) were generally inversely correlated. Each arrow represents a test subject: GP.Mur athletes in black color, non-GP.Mur athletes in dark gray color, and non-GP.Mur non-athletes in light gray color. The beginning of an arrow indicated concentrations of plasma nitrite and nitrate right before cuffing; the arrowhead indicated concentrations of plasma nitrite and nitrate upon release of the cuff pressure. RIGHT: Plasma nitrite generally increased upon cuff deflation following 5-min arterial occlusion. Each line connected the nitrite concentration before arm cuffing and the concentration upon pressure deflation for an individual subject. **(B)** The concentrations of blood plasma nitrite were directly proportional to individual band 3 levels. The thick gray line indicated significant linear fitting for non-GP.Mur (*p < 0.05); the thin dashed line indicated poor linear fitting for GP.Mur data (*n.s*.). LEFT: the concentration of plasma nitrite ([${\text{NO}}_{2}^{-}$]) before cuffing. RIGHT: [${\text{NO}}_{2}^{-}$] upon cuff deflation following 5-min occlusion of the brachial artery. **(C)** For all non-GP.Mur subjects (athletes and non-athletes), their changes of blood nitrite concentrations by arterial occlusion during FMD (Δ[${\text{NO}}_{2}^{-}$]_cuffing_ = [${\text{NO}}_{2}^{-}$]_pre−cuff_ - [${\text{NO}}_{2}^{-}$]_upon deflation_) was directly correlated with erythroid band 3 levels (*p < 0.05). This direct relation between band 3 and (Δ[${\text{NO}}_{2}^{-}$]_cuffing_) was not apparent in GP.Mur+ subjects.

Similar to the FMD/NOx study by Allen et al. (31), we observed that occlusion of the brachial artery followed by cuff deflation decreased systemic plasma nitrate and increased systemic plasma nitrite contents (Table 2, Figure 4A). But the concentrations of plasma nitrite, nitrate, or NOx before pressure cuff or right after cuff deflation were not different between GP.Mur+ and non-GP.Mur athletes (Table 2). The increase of nitrite or the decrease of nitrate by arterial occlusion and cuff release (Δ[${\text{NO}}_{2}^{-}$]_cuffing_ & Δ[${\text{NO}}_{3}^{-}$]_cuffing_) were also not significantly different between GP.Mur and non-GP.Mur (Table 2). The level of plasma nitrite is a balance between nitrite production and nitrite consumption. Under physiologically oxygenated conditions, most nitrite is constitutively generated in tissue from oxygenation of NO produced by eNOS (34), and is consumed by reduction into NO primarily via deoxy Hb (35). Individual eNOS levels were expected to vary, and hence individuals with high [${\text{NO}}_{2}^{-}$]_pre−FMD_ generally showed high [${\text{NO}}_{2}^{-}$]_upon deflation_ (Figure 4B).

Band 3 complexes have been suggested to assist Hb in NO-dependent hypoxic vasodilation, either as a nitrite transporter (7) or/and as a mediator for permeation of NOx/SNO metabolites across the red cell membrane (8, 9). Band 3 expression was slightly higher in people with GP.Mur (Table 2) (18, 22, 36). We thus explored the possible involvement of band 3 in NOx changes during FMD. The concentrations of systemic plasma nitrite before cuff pressing and after deflation were both directly correlated to band 3 expression levels on the RBC membrane (Figure 4B). The correlation between band 3 and systemic nitrite was better before than after regional arterial occlusion (Figure 4B: larger Pearson's correlation *r* before cuffing [left] than after cuff deflation [right]). Notably, for GP.Mur+ athletes, their band 3-nitrite correlation was much smaller and diminished after cuff deflation. We calculated the extent of systemic nitrite changes induced by this regional hypoxia ([${\text{NO}}_{2}^{-}$]_post_ - [${\text{NO}}_{2}^{-}$]_pre_, or Δ[${\text{NO}}_{2}^{-}$]_cuffing_), which was significantly correlated with erythroid band 3 levels but only in non-GP.Mur subjects (Figure 4C). This discrepancy suggests different impacts of GP.Mur+ and non-GP.Mur membrane structure on NOx metabolism, as seen in Figure 3A.

## Discussion

Intraerythrocytic Hb is the major NO scavenger inside human body, but deoxy Hb as a nitrite reductase also generates NO in low O_2_ during the respiratory cycle. The sources of NO for maintaining the vascular tone mainly come from constitutive conversion of L-Arginine by endothelial NO synthase (eNOS) and from reduction of NOx by heme reductase like deoxy Hb. NO production by deoxy Hb complements NO production by eNOS, which takes place preferentially in high O_2_. This may explain, at least in part, why eNOS expression alone cannot fully reflect NO bioavailability or FMD (37–39).

Upon deflation of the cuff pressure in FMD, we observed decrease of plasma nitrate and increase of plasma nitrite for both GP.Mur and non-GP.Mur groups (Table 2, Figure 4A). The reaction of NO in oxygenated RBCs mainly forms nitrate; the reaction of NO in oxygenated tissues mainly forms nitrite. Thus, decreasing nitrate suggests lower NO and a need to increase NO/nitrite bioavailability from both tissues and RBCs. Nitrite synthesis was accelerated to increase NO bioavailability.

**Figure 5:**
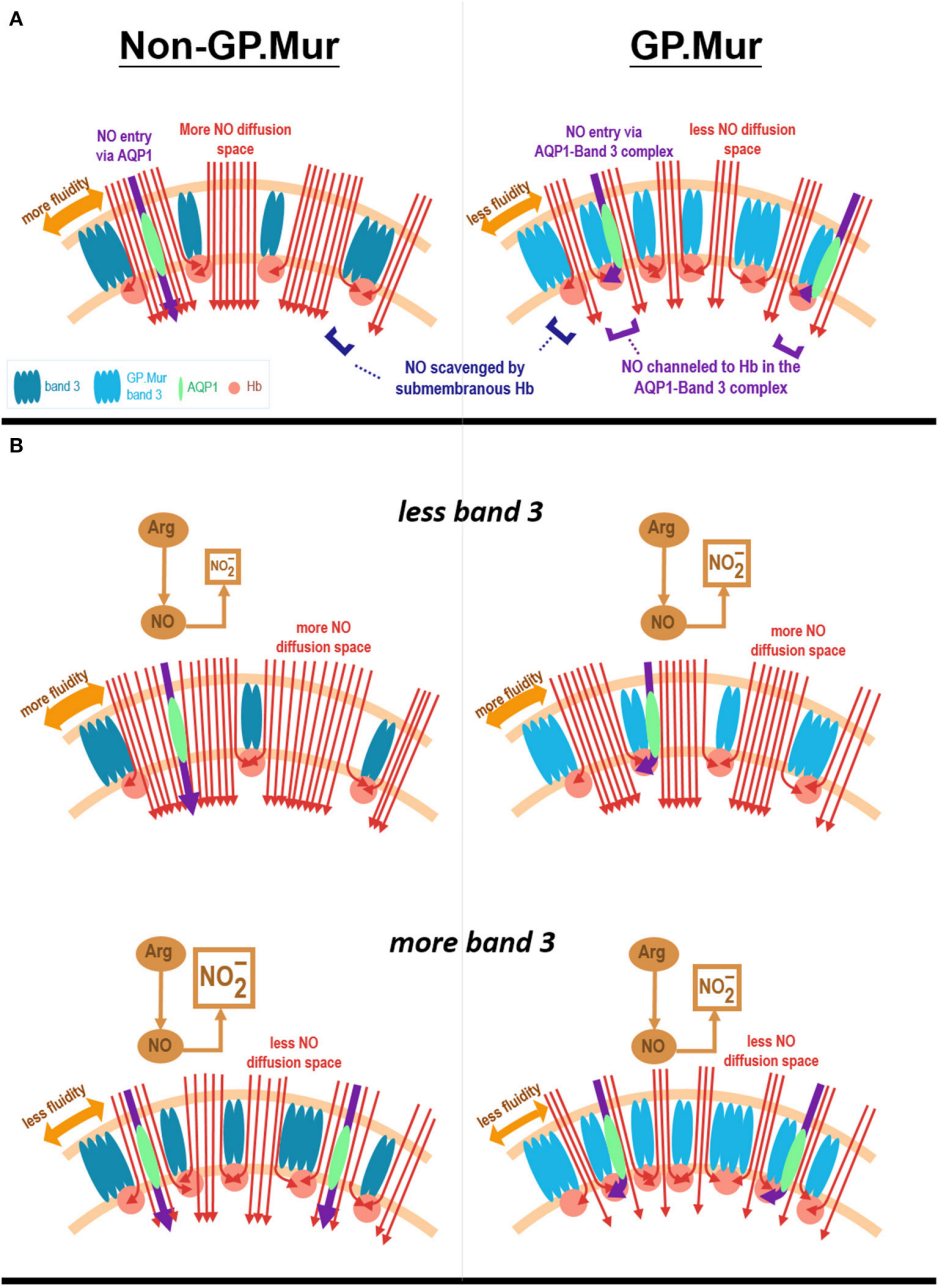
Hypothesis. **(A)** A model depicts how Hb in GP.Mur+ RBCs could be more effective in scavenging NO than Hb in non-GP.Mur RBCs. GP.Mur band 3 dimers and tetramers differ from non-GP.Mur in higher expression and distinct structural organization (labeled with different shades of blue color). Cytoplasmic band 3 binds Hb (red balls), preferentially less O_2_-saturated Hb. Because GP.Mur+ RBCs express more band 3, there is more Hb lining on the inner leaflet of the lipid bilayer. In terms of protein complex organization, AQP1 (green) is mobile in non-GP.Mur RBCs but is often associated with band 3 in GP.Mur+ RBCs. Their oligomerization on the GP.Mur+ membrane enables channeling of NO to Hb through the AQP1-band 3 complexes (shown in purple arrows). Both structural differences equip GP.Mur+ RBCs with a stronger suction for NO (red arrow lines). **(B)** A model depicts the relations between erythroid band 3 expression and plasma nitrite levels. Band 3 expression on the RBC membrane vary among individuals. The red cell membrane inserted with more band 3 protein tends to be less fluid and hence allows for less NO diffusion into the red cells (shown in red arrow lines). For non-GP.Mur (left panels), higher band 3 expression is associated with less NO entry and scavenge by erythrocytes; their plasma nitrite levels are thus higher. The content of plasma nitrite is complementary to the amount of NO scavenged by RBCs. For GP.Mur (right panels), NO diffusion into erythrocytes is not only affected by band 3 protein expression/membrane fluidity; GP.Mur+ RBCs also scavenge NO more efficiently (as explained in **A**). For GP.Mur, these two opposite effects conceivably even out and obscure the direct correlation between erythroid band 3 and plasma nitrite levels that was seen in non-GP.Mur.

This study probed into the effects of GP.Mur/band 3 on NO-dependent vasodilation. Our age-matched, young elite athletes all had similar blood pressure, but GP.Mur+ athletes had already exhibited lower FMD than non-GP.Mur counterparts (Figure 2). Their Hb levels were all within the normal range, but Hb in GP.Mur+ RBCs was more effective in limiting NO-dependent vasodilation (Figures 2, 3). This suggests that GP.Mur/band 3-associated structural features could further facilitate NO scavenge by intraerythrocytic Hb (Figure 3A). Band 3 protein complexes differ between GP.Mur and non-GP.Mur in two aspects: (1) there are more band 3 molecules on the GP.Mur+ RBCs; (2) GP.Mur+ band 3 complexes are structurally distinguishable from non-GP.Mur (18, 25–27). In particular, AQP1 and band 3 interact more strongly in GP.Mur+ than in non-GP.Mur RBCs (26). The water channel AQP1 is also a NO gas channel (40–42). Thus, we hypothesize that Hb in GP.Mur+ RBCs was more effective in scavenging NO for the two reasons: (1) more band 3 on the GP.Mur+ membrane could attract more Hb to the inner leaflet of the cell membrane; (2) their stronger AQP1-band 3 interaction could channel NO influx via AQP1 to band 3-bound Hb (Figure 5A). Though Hb was not different between GP.Mur+ and non-GP.Mur groups (Table 1), more Hb distributed on the inner leaflet would result in more efficient NO scavenge. Since NO gas diffuses into erythrocytes extremely rapidly (at a rate near its diffusion limit), the rate of NO scavenge by intracellular Hb is critical to NO bioavailability and NO-dependent vasodilation (as in Figure 3A).

We also found that erythroid band 3 levels were directly correlated with Δ[${\text{NO}}_{2}^{-}$]_FMD_ in non-GP.Mur but not in GP.Mur+ subjects (Figure 4). Band 3 likely could support permeation of ${\text{NO}}_{2}^{-}$ across the red cell membrane, as proposed by other groups (7, 8). Band 3 transports ${\text{HCO}}_{3}^{-}$ at the rate of ~10^4^/s. If band 3 transports ${\text{NO}}_{2}^{-}$ at a similar rate as ${\text{HCO}}_{3}^{-}$, this is still much slower than the rate of NO diffusion through the plasma membrane. Thus, the different correlations between band 3 levels and Δ[${\text{NO}}_{2}^{-}$]_FMD_ more likely involved different degrees of NO scavenge between GP.Mur and non-GP.Mur.

We hypothesize that for non-GP.Mur RBCs, a higher band 3 protein level shall decrease membrane fluidity and hence reduce NO diffusion into the red cells (Figure 5B, bottom). This is because the inner leaflet of the RBC membrane is mainly covered by metHb. Despite that metHb in RBCs is only 0.5–3%, it reacts with NO at a much slower rate than oxyHb or deoxyHb, and thus forms a barrier for NO diffusion into the red cells. Increase of band 3 expression in non-GP.Mur RBCs thus could reduce NO entry and scavenge; more plasma nitrite could then be observed (Figure 5B). On the other hand, the lack of the correlation between erythroid band 3 and plasma nitrite levels in GP.Mur+ RBCs suggest that factors other than membrane barriers to NO diffusion (i.e., metHb and fluidity) were involved. These other factors include more extensive lining of Hb with cytoplasmic GP.Mur band 3 in the inner leaflet, and unique channeling of NO to submembranous Hb via AQP1-band 3 complexes (Figure 5A). Because of these additional factors, higher band 3 levels in GP.Mur+ RBCs conceivably also promote NO scavenging. These effects were opposite to the decrease of NO entry in less fluid red cells (Figure 5B). These may explain why the direct correlation between plasma nitrite and band 3 levels seen in non-GP.Mur subjects was absent in GP.Mur (Figures 4, 5).

A final interesting observation from this study is the lower plasma nitrate (but not nitrite) among the elite athletes, regardless of GP.Mur (Table 2). Skeletal muscles are the dominant nitrate reservoir over other organs or blood (43). It has been reported that the nitrate stores, particularly that in skeletal muscles, are essentially reduced to nitrite and NO for vasodilation during high-intensity exercise (43). Our athlete subjects were routinely trained at high intensity. Indeed, their nitrate store was more depleted than that in the non-athlete controls (Table 2). Thus, from our results, even young athletes will need nitrate supplementation more than non-athletes of similar age, not just for sports performance but also for their vascular health (43).

### Limitation

This work nonetheless is limited by the small sample size for a FMD study (44). The main reason is the low prevalence of GP.Mur blood type in northern Taiwan (<2%) where this study was conducted. To minimize variations among subjects, we recruited only male elite athletes from top sports universities in the region. But this type of study may be even harder to conduct in the future, as we have noticed gradual dilution of the *GYP.Mur* gene in Taiwan in the past half century.

## Data Availability Statement

The raw data supporting the conclusions of this article will be made available by the authors, without undue reservation.

## Ethics Statement

The studies involving human participants were reviewed and approved by MMH-Institutional Review Board (IRB registration: 19MMHIS081e). The patients/participants provided their written informed consent to participate in this study.

## Author Contributions

KH, Y-YL, and W-CT conceived the project. W-CT and K-WT recruited the subjects. Y-YL, KH, and H-JL performed ultrasound experiments. C-YL supported ultrasound experiments. L-YC, H-LL, and H-JL performed blood tests and analyzed the data. K-TH helped conceptualize the data. H-IY edited the paper. KH analyzed the data and wrote the paper. All authors contributed to the article and approved the submitted version.

## Funding

This work was supported by grants from Taiwan Ministry of Science & Technology (MOST 108-2628-B-195-001; MOST 109-2628-B-195-001; MOST 110-2628-B-195-001), and MacKay Memorial Hospital to KH (MMH 108-149; MMH 109-21; MMH 110-25; MMH 111-26, TTMMH 107-06; TTMMH 109-04).

## Conflict of Interest

The authors declare that the research was conducted in the absence of any commercial or financial relationships that could be construed as a potential conflict of interest.

## Publisher's Note

All claims expressed in this article are solely those of the authors and do not necessarily represent those of their affiliated organizations, or those of the publisher, the editors and the reviewers. Any product that may be evaluated in this article, or claim that may be made by its manufacturer, is not guaranteed or endorsed by the publisher.
